# Comprehensive assessment of markers of apoptosis and cell proliferation during progression of atherosclerosis after surgery in patients with peripheral arterial disease

**DOI:** 10.1590/1677-5449.202200292

**Published:** 2023-02-10

**Authors:** Roman Evgenyevich Kalinin, Igor Aleksandrovich Suchkov, Emma Anatolievna Klimentova

**Affiliations:** 1 Ryazan State Medical University, Ryazan, Russia.

**Keywords:** atherosclerosis, apoptosis, vascular wall, cell proliferation, Вах, sFas, aterosclerose, apoptose, parede vascular, proliferação celular, Вах, sFas

## Abstract

**Background:**

Determination of predictors that can affect development of atherosclerosis progression in the postoperative period is an urgent problem in vascular surgery.

**Objectives:**

Integrated assessment of markers of apoptosis and cell proliferation in atherosclerotic lesions and their progression after surgery in patients with peripheral arterial diseases.

**Methods:**

The investigation included 30 patients with stage IIB-III peripheral arterial disease. All patients have undergone open surgical interventions on the arteries of the aorto-iliac and femoral-popliteal segments. During these interventions, intraoperative specimens were obtained from the vascular wall with atherosclerotic lesions. The following values were evaluated: VEGF А165, PDGF BB, and sFas. Samples of normal vascular wall were obtained from post-mortem donors and used as a control group.

**Results:**

The levels of Bax and p53 were increased (p<0.001) in samples from arterial wall with atherosclerotic plaque, while sFas values were reduced (p<0.001), compared to their levels in control samples. Values of PDGF BB and VEGF A165 were 1.9 and 1.7 times higher in atherosclerotic lesion samples (p=0.001), in comparison with the control group. The levels of p53 and Bax were increased against a background of reduced sFas levels in samples with progression of atherosclerosis compared to their baseline values in samples with atherosclerotic plaque (p<0.05).

**Conclusions:**

Initially increased values of the Bax marker against a background of reduced sFas values in vascular wall samples from patients with peripheral arterial disease is associated with risk of atherosclerosis progression in the postoperative period.

## Resumo

**Contexto:**

A determinação de preditores que possam influenciar o desenvolvimento da progressão da aterosclerose no período pós-operatório é um problema urgente em cirurgia vascular.

**Objetivos:**

Realizar uma avaliação integral de marcadores de apoptose e proliferação celular nas lesões ateroscleróticas e sua progressão após cirurgia em pacientes com doenças arteriais periféricas.

**Métodos:**

A investigação incluiu 30 pacientes com doenças arteriais periféricas de estágio IIB-III. Todos os pacientes foram submetidos a intervenções operatórias abertas nas artérias dos segmentos aorto-ilíaco e fêmoro-poplíteo. Durante a intervenção, foi obtido material intraoperatório da parede vascular com lesões ateroscleróticas. Foram avaliados os seguintes valores: VEGF A165, PDGF BB e sFas. Como grupo controle, amostras de parede vascular normal foram obtidas de doadores *post-mortem*.

**Resultados:**

O nível de Bax e p53 (p < 0,001) em amostras de parede arterial com placa aterosclerótica estava elevado em meio a valores reduzidos de sFas (p < 0,001) em comparação ao grupo controle. Os valores de PDGF BB e VEGF A165 foram 1,9 e 1,7 vezes maiores, respectivamente, nas amostras com lesão aterosclerótica (p = 0,001) do que no grupo controle. O nível de Bax e p53 e Bax estava elevado no contexto de nível reduzido de sFas em amostras com progressão da aterosclerose em comparação com seus valores basais em amostras com placa aterosclerótica (p < 0,05).

**Conclusões:**

Níveis inicialmente elevados do marcador Bax no contexto de valores reduzidos de sFas na parede vascular em pacientes com doença arterial periférica estão associados a risco de progressão da aterosclerose no período pós-operatório.

## INTRODUCTION

Peripheral arterial disease (PAD) is one of the leading causes of disability and mortality among vascular diseases.^1^ One of the most effective methods of treating patients with PAD is considered to be open vascular reconstructions of arteries of the lower extremities.^2^ However, atherosclerosis progression and restenosis of the reconstruction zone may require additional re-interventions which increase the risk of limb loss.^3^

The processes underlying development of progression of atherosclerosis and restenosis of the intervention zone in the postoperative period are still incompletely understood.^4^

Modern studies consider involvement of the apoptosis system in the development of atherosclerosis.^5^ In our previous articles, we have managed to prove the influence of the apoptosis system on development of restenosis of the reconstruction zone.^6^,^7^ At the same time, we have not found any work devoted to studying the effect of cell death on development of atherosclerosis progression after vascular reconstructions of the arteries of the lower extremities. The primary biomarkers of the apoptosis system include the pro-apoptotic marker - Bcl-2-associated X protein (Bax); the anti-apoptotic marker- B-cell lymphoma 2 (Bcl-2); the inhibitor of the apoptosis receptor pathway, the soluble form of the Fas receptor (sFas); and the p53 protein.^8^,^9^

The apoptosis system is linked to the processes of proliferation and migration of vascular wall cells. Therefore, from a physiological point of view, it is more appropriate to consider these processes together.^10^ The main marker responsible for proliferation and migration of smooth muscle cells of the vascular wall is platelet growth factor - PDGF BB - while for endothelial cells it is vascular endothelial growth factor - VEGF A165.^11^

As a result of our primary studies, a number of hypotheses were put forward, specifically, with regard to the relationship between biomarkers of apoptosis and cell proliferation and the possibility that these markers could be predictors of the progression of atherosclerosis after open interventions on the arteries of the lower extremities.

## OBJECTIVES

To conduct an integrated assessment of markers of apoptosis and cell proliferation in atherosclerotic lesions and their progression after surgery in patients with peripheral arterial diseases.

## PATIENTS AND METHODS

A prospective cohort study recruited 30 patients with stage IIB-III PAD according to the Fontaine classification (Rutherford category 3-4), who were allocated to group A (G A). All participants signed free and informed consent forms after being given information about the study. The average age of patients was 65 [61; 69], the number of men was 18 (60%). The number of patients with stage IIB disease was 14 (46%), while 16 had stage III disease (54%). Patients’ comorbidities were as follows: ischemic heart disease (25%); hypertension (44%); and obstructive pulmonary disease (29%).

The inclusion criteria were males or females aged ≥40 years old and presence of PAD.

Criteria for exclusion from the study were chronic ischemia of the lower extremities of another etiology; active cancer or remission period of less than 5 years; presence of diabetes mellitus; and a history of vascular interventions on the arteries of the lower extremities. After additional examination, all patients underwent open reconstructive surgery on the arteries of the aortoiliac and femoral-popliteal segments. The different types of operations performed are listed in Table 1.

**Table 1:** Types of surgery in group А patients.

**Types of surgery**	**Number of patients, n (%)**
Aortobifemoral bypass	7 (23.3%)
Femoro-popliteal bypass above the knee, using a synthetic prosthesis	14 (46.7%)
Femoral-popliteal bypass above the knee, using an autologous vein	8 (26.6%)
Femoral-femoral crossover bypass	1 (3.4%)

Intraoperative specimens were collected during operations including all three layers of a vascular wall segment with an atherosclerotic lesion. Sampling areas were the common iliac artery, the common femoral artery, and the superficial femoral artery. According to the Stary-classification, the degree of atherosclerosis assessed was Vb (advanced lesions). A section of the vessel was separated with a scalpel and 0.15 g of tissue was weighed. Samples were ground and homogenate was prepared using a Thermo Fisher Scientific (USA) lysis buffer in a DIAX 900 (Heidolph, Germany) (6G nozzle) high-speed rotary homogenizer at a speed of 24000 rpm for 60 sec at a temperature of +2 °C. The resulting homogenate was centrifuged at 1000 g for 10 minutes (t +2 °C). Levels of Bax; Bcl-2; p53; sFas; VEGF A165; and PDGF BB proteins in the resulting supernatant were assessed using enzyme immunoassay. In the resulting supernatant, levels of VEGF A165; PDGF BB; sFas; p53; and Bcl-2 proteins were determined using “Invitrogen Thermo Fisher” commercial kits (USA), while Bax - (Bcl-2 Associated X Protein) was assayed using a “Cloud-Clone Corporation” kit (China, USA). The indicators obtained were recalculated for protein content, which was estimated by the Bradford method using the Coomassie Plus (Bradford) Assay Kit (Thermo Fisher Scientific, USA).

**Figure 1:**
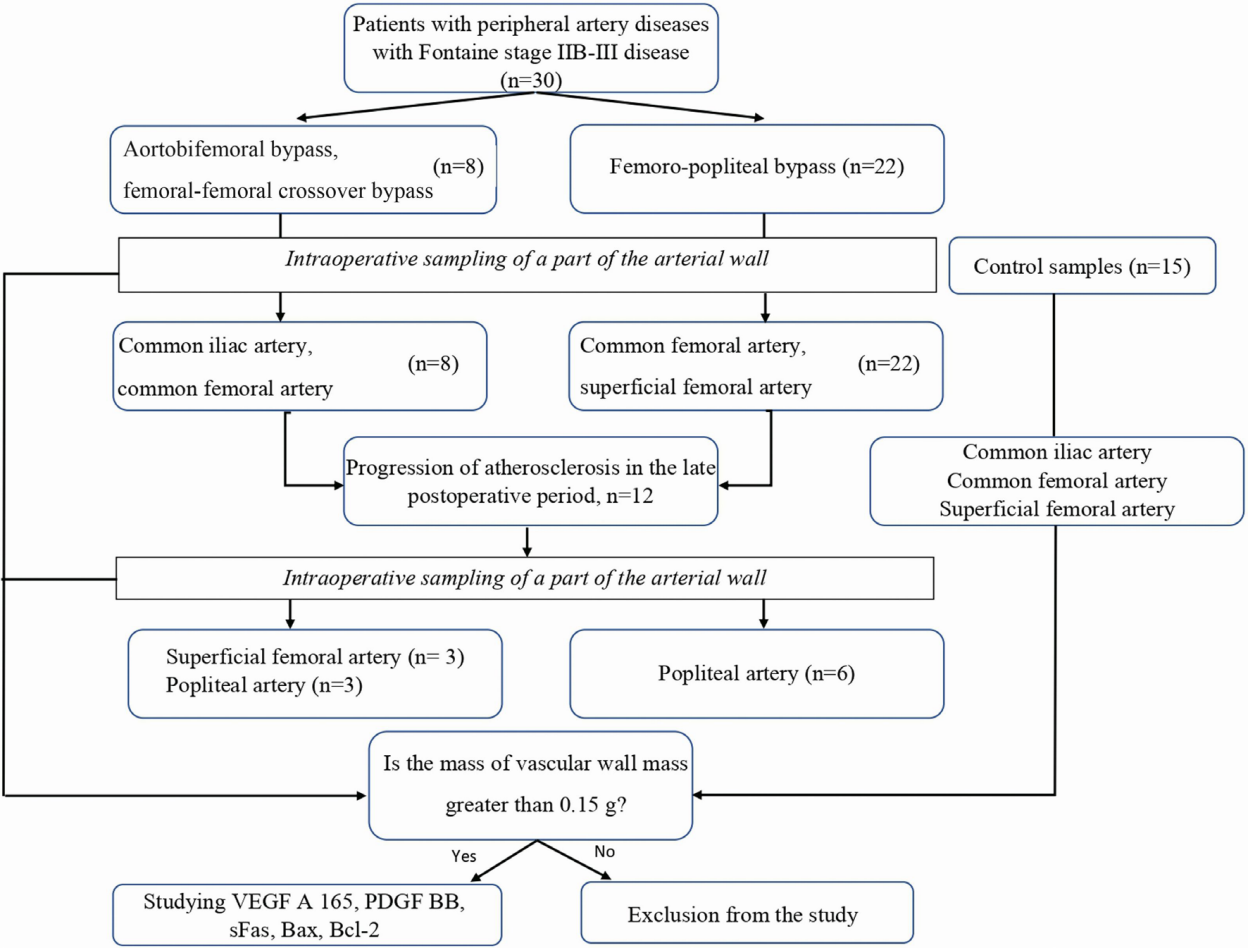
Scheme for including samples of the vascular wall in the study.

**Figure 2:**
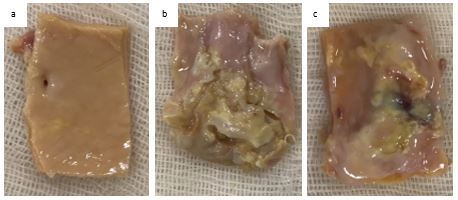
Vascular wall samples. Note: (a) samples with normal arterial wall; (b) samples with atherosclerotic lesions; (c) samples with progression of atherosclerosis.

To determine reference values of the biomarkers studied, samples of the arterial wall were taken during explanation of organs from post-mortem donors without atherosclerotic lesions (according to duplex scanning [DS] of the arteries of the lower extremities) during multivisceral sampling, and allocated to group B (G B). There were 15 samples. All donors were male, the mean age of patients was 59 [49; 62] years. The sampling areas were the common iliac artery, the common femoral artery, and the superficial femoral artery (Figures 1 and 2). In terms of age and sex composition, patients in G B were comparable to the patients in G A (p>0.05). These vascular wall samples were conditioned in a solution of custadiol with the addition of gentamicin (400 mcg/mL) and fluconazole (20 mcg/mL) at a temperature of +40 °C and prepared under working conditions for use as material for the preparation of vascular wall homogenate.

The follow-up period for G A patients was 2 years. During this period, patients were under dynamic observation with DS of the arteries of the lower extremities at 6, 12, and 24 months in order to detect any progression of atherosclerosis. Progression of atherosclerosis was observed after 23 [22-24] months and was defined as any increase in the degree of stenosis outside the revascularization zone or any *de novo* atherosclerotic lesion detected. On this basis, group A patients were divided into two subgroups: subgroup A1 (G A1) - patients with progression of atherosclerosis during the long-term postoperative period (n=12), and subgroup A2 (G A2) - patients without this complication (n=18). Since recurrent limb ischemia developed due to progression of atherosclerosis in the G A1 patients after their primary operations, repeat interventions were performed and specimens were taken of the arterial wall with foci of atherosclerosis progression. Subsequently, levels of the markers studied were evaluated in these samples. Thanks to a carefully developed technique of sampling, transportation of the vascular wall in patients with PAD, and subsequent preparation of the homogenate, it was possible to immediately include 30 patients with no losses. During the 2-year follow-up period, no patients refused to participate in the study and there were no deaths.

The Miot^12^ and Narkevich and Vinogradov^13^ methodology for calculating the minimum size of independent samples for quantitative indicators was used to determine the minimum number of patients required to identify significant differences between the indicators of apoptosis and cell proliferation. The study compared two unrelated groups: G A - patients with atherosclerotic lesions of the vascular wall (n1) and G B - patients with a normal arterial wall (n2). According to the results of this calculation, the minimum number of patients for the study was 22 patients: n1=12, n2=10.

The critical level for statistical significance is p<0.05. Study power β = 0.8. The Kolmogorov-Smirnov test was used to determine the shape of data distributions (p<0.05). Due to deviation from the Gaussian distribution, the Mann-Whitney test was used to compare the values from unrelated groups (comparison of the values of markers of apoptosis and cell proliferation in vascular wall samples taken from patients from G A and G B; G B and G A1; comparison of biomarkers in the vascular wall during progression of atherosclerotic lesions and their baseline values in atherosclerotic plaques from patients in G A1).

Correlation analysis of samples was performed using the Spearman test.

Roc-analysis with addition of Roc curves was used to find threshold values for sFas and Bax markers; values of markers above/below these cutoffs are associated with the risk of atherosclerosis progression. Data are presented as median and 1st and 3rd quartiles (Me [Q1; Q3]). Statistical processing was carried out using SPSS Statistics 26.0 (IBM company, USA) and Microsoft Excel 2019.

This study was approved by the local ethics committee at the Ryazan State Medical University named after Academician I.P. Pavlova (extract No. 7, dated 03.03.2020).

## RESULTS

It was found that the amount of pro-apoptotic Bax protein was increased by 48% in samples of the arterial wall with atherosclerotic lesions from patients in G A compared to its value in control samples from G B (p<0.001), while differences in the marker Bcl- 2 in the groups compared were not significant (p=0.138). We should also highlight that the level of p53 protein was increased by 55% (p<0.001) and the amount the sFas inhibitor of the apoptosis receptor pathway was reduced by 82% (p=0.001) in samples of vascular wall with atherosclerotic lesions from G A patients compared with samples from G B. In turn, the values of the PDGF BB and VEGF A165 markers of proliferation and migration were also increased by 1.9 and 1.8 times, respectively, in samples with atherosclerotic plaque from G A patients (p = 0.001), compared with samples from G B patients, Table 2.

**Table 2:** Comparison of values of the biomarkers studied in the vascular wall of patients in groups A and B.

**Indicators Me [Q1-Q3]**	**Р53 (u/mg protein)**	**PDGF ВВ (ng/mg protein)**	**Всl-2 (ng/mg protein)**	**Вах (ng/mg protein)**	**sFas (ng/mg protein)**	**VEGF А 165 (pg/mg protein)**
G B						
Samples with normal arterial wall	0.19	0.09	1.0	4.8	0.3	8.1
	[0.17; 0.22]	[0.07; 0.12]	[0.9; 1.2]	[3.8; 6.3]	[0.24; 0.39]	[6.8; 9.3]
G A						
Samples with atherosclerotic lesions	0.29*	0.18*	0.9	7.1^*^	0.17*	14.5*
	[0.26; 0.34]	[0.1; 0.3]	[0.64; 1.2]	[6.5; 7.6]	[0.1; 0.2]	[12; 18]

* Statistically significant difference, p <0.05.

The results of the correlation analysis of the biomarkers studied revealed an inverse relationship between the sFas marker and the proapoptotic marker Bax (r= -0.938, p<0.001) in samples of the vascular wall with atherosclerotic lesions from G A patients. Moreover, markers VEGF A165 and PDGF BB (r=+0.561, p<0.001); Bcl-2 and PDGF BB (r=+0.594, p<0.001); and VEGF A165 and Bcl-2 (r=+0.441, p<0.001) were directly correlated with each other.

Primary patency in G A patients was 60% 2 years after surgery. In 12 (40%) patients, 23 [22-24] months after surgery, progression of atherosclerotic lesions occurred in the operated limb, with development of critical ischemia (G A1). It should be noted that patients in G A1 were comparable with patients without progression of atherosclerosis in terms of initial angiological status, type of surgical intervention, and comorbidity (G A2, p>0.05) (see the Supplementary Material).

One of the stages of the study included analysis of the values of the markers studied in the samples of arterial wall taken directly from the zone of progressive atherosclerotic lesions during repeat arterial reconstructions in G A1 patients. These samples had 2.1 times greater values for p53 markers (p=0.002) and Bax values were 1.9 times greater (p<0.01), compared with their values in G B control samples. The levels of the PDGF BB and VEGFA165 markers of proliferation and migration of vascular wall cells were increased by 2.3 and 2 times (p<0.01), respectively, compared with their values in G B control samples. The level of the apoptosis receptor pathway inhibitor sFas was reduced by 4.6 times (p<0.01, Table 3).

**Table 3:** Comparison of the values of the biomarkers studied in the vascular wall of patients in groups A1 and B.

**Indicators Me [Q1-Q3]**	**Р53 (u/mg protein)**	**PDGF ВВ (ng/mg protein)**	**Всl-2 (ng/mg protein)**	**Вах (ng/mg protein)**	**sFas (ng/mg protein)**	**VEGF А165 (pg/mg protein)**
G B						
Samples with normal arterial wall	0.19	0.09	1.0	4.8	0.3	8.1
	[0.17; 0.2]	[0.07; 0.12]	[0.9; 1.2]	[3.8; 6.3]	[0.24; 0.39]	[6.8; 9.3]
G A1						
Samples with progression of atherosclerosis	0.41^*^	0.21*	0.82	9.3*	0.06*	16.5*
	[0.38; 0.48]	[0.18; 0.29]	[0.7; 0.9]	[8.8; 10.3]	[0.05; 0.09]	[15; 18.5]

* Statistically significant difference, p <0.05.

The results of the correlation analysis revealed relationships between proapoptotic markers and the levels of proliferation and migration of smooth muscle cells of the vascular wall, namely, Bax and PDGF BB (r=-0.646, p=0.023); p53 and PDGF BB (r=-0.626, p= 0.03); and sFas and PDGF BB (r=-0.828, p <0.01) in samples with progression of atherosclerosis in G A1patients.

**Figure 3:**
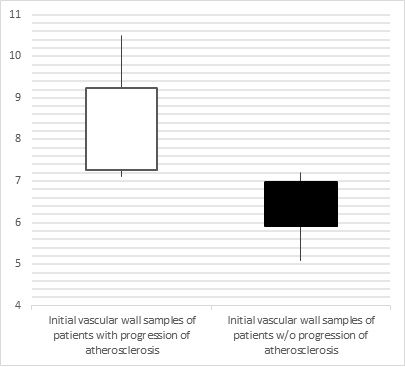
Comparison of the initial values of the marker Bax in the vascular walls of patients with progression of atherosclerosis and patients without this complication.

**Figure 4:**
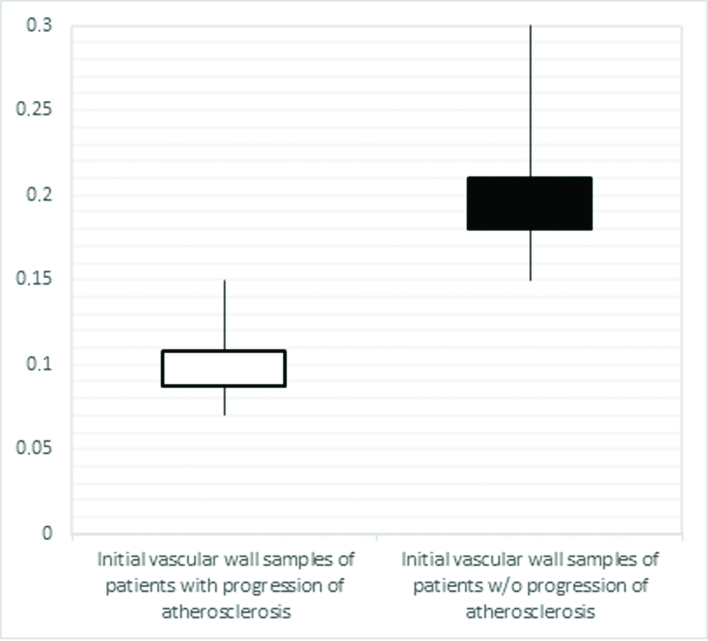
Comparison of the initial values of the marker sFas in the vascular walls of patients with progression of atherosclerosis and patients without this complication.

Comparison of the initial baseline values for biomarkers in the vascular wall in the area of atherosclerotic lesions from patients with progression of atherosclerosis (G A1) to the values from patients without this outcome (G A2) revealed the following differences: the level of the Bax marker was increased 1.3 times, while the sFas level was reduced 1.9 times in G A1 patients compared with G A2 patients (p<0.001, Figures 3 and 4).

**Figure 5:**
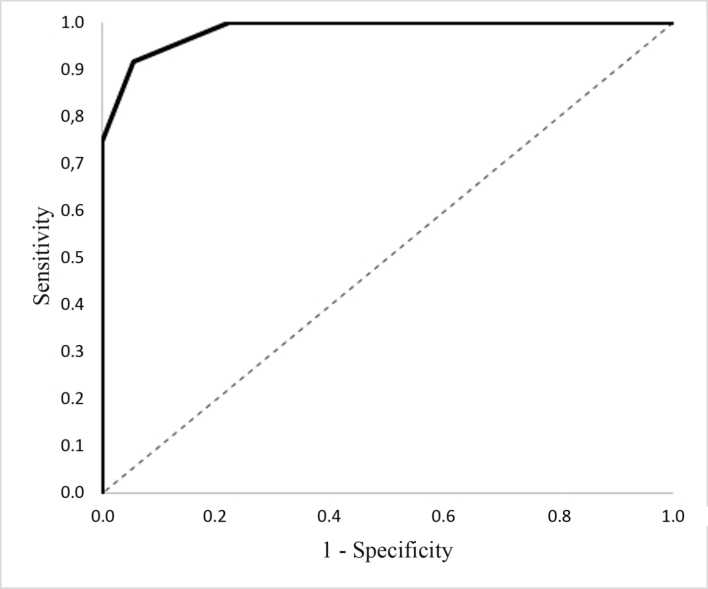
Influence of the initial values of the marker Bax in the vascular wall in the area of atherosclerotic lesions on the progression of atherosclerosis in the postoperative period.

Roc-analysis performed on the Bax marker from initial baseline arterial samples from atherosclerotic lesions in G A1 patients showed that the prognostic cut-off value is 7.15 ng/mg protein (AUC = 0.98, 95% CI: 0.95-1; p=0.001), while the sensitivity and specificity of this method were 91.7% and 94.4%, respectively (Figure 5).

**Figure 6:**
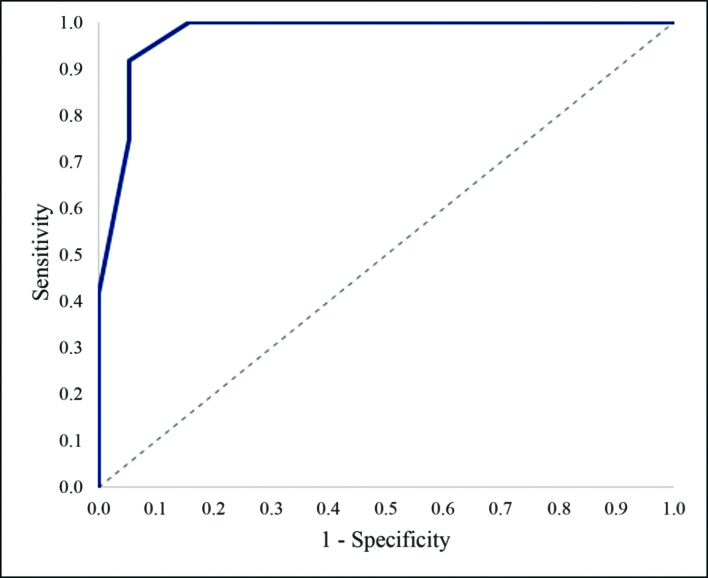
Influence of the initial values of the sFas marker in the vascular wall in the area of atherosclerotic lesions on the progression of atherosclerosis in the postoperative period.

For the sFas marker, the threshold value at the cut-off point, determined using the Youden index, is 0.14 ng/ mg protein (AUC 1.0, 95% CI: 0.98-1.0, p<0.001). Disease progression is predicted when the initial value of sFas in the vascular walls in the area of atherosclerotic plaque is equal to or below this cut-off point. The sensitivity and specificity of this method are 91.7% and 100% respectively (Figure 6).

Subsequently, the initial baseline values of the parameters studied in samples of the vascular wall with atherosclerotic lesion were compared with their levels in samples taken after progression of atherosclerosis in G A1 patients, obtaining the following results. Levels of p53 and Bax biomarkers were significantly increased against the background of reduced sFas values in samples with progression of atherosclerosis compared to their initial values in samples with atherosclerotic lesions (p<0.05). Parameters of PDGF BB, VEGF A165 and Bcl-2 in comparable samples did not differ significantly (p>0.05, Table 4).

**Table 4:** Comparison of the biomarkers studied in the vascular wall during the progression of atherosclerotic lesions and their initial values in atherosclerotic plaque in group A1 patients

**Indicators Me [Q1-Q3]**	**Р53 (u/mg protein)**	**PDGF ВВ (ng/mg protein)**	**Всl-2 (ng/mg protein)**	**Вах (ng/mg protein)**	**sFas (ng/mg protein)**	**VEGF А 165 (pg/mg protein)**
Samples with atherosclerotic plaque	0.3	0.18	0.85	8.3	0.1	15.6
	[0.28; 0.34]	[0.1; 0.2]	[0.64; 1.2]	[7.3; 9.4]	[0.08; 0.12]	[12.5; 19]
Samples with progression of atherosclerosis	0.41^*^	0.21	0.82	9.3*	0.06*	16.5
	[0.38; 0.48]	[0.18; 0.29]	[0.7; 0.9]	[8.8; 10.3]	[0.05; 0.09]	[15; 18.5]

* Statistically significant difference, p <0.05.

## DISCUSSION

Despite the fact that the number of surgical interventions performed on the arteries of the lower extremities is increasing, identifying predictors that can determine the possibility of a successful operation in the long term remains an urgent problem. The relative role of pathobiological processes such as growth, death, cell migration, matrix modification, and vascular wall remodeling remains a matter for debate and ongoing research.

The results of our work based on the set goal of the study showed that all of the biomarkers of the system of apoptosis and cell proliferation studied were detected in the intact arterial wall. This may be due to the fact that normally the cells of the vascular wall undergo apoptotic death, which is the possibility of removing infiltrating leukocytes and damaged cells without development of inflammation. In this case, the natural death of cells is compensated by mitogenesis of new cells, which is under the control of growth factors.

In atherosclerotic lesions of the vascular wall, all proapoptotic markers of two apoptosis pathways, namely Bax and p53, were elevated, while there was a reduced level of the apoptosis receptor pathway inhibitor sFas. There are a number of studies considering pro-inflammatory interleukins, oxidized lipoproteins, hypoxia, and oxidative stress from the perspective of cell apoptosis triggers in atherosclerotic plaques.^14^,^15^ Bian W, in his study, showed that most of the apoptotic cells are localized in areas rich in activated T-lymphocytes and macrophages. In our study, in addition to an increase in the number of apoptosis markers, increases were also found in the values of PDGF BB and VEGF A165 markers. The joint presence of growth factors (PDGF BB and VEGF A165) and cell death in atherosclerotic plaques can be explained as follows: two processes, namely apoptosis and proliferation are interrelated and can regulate each other. The correlation revealed between Bcl-2, VEGF A165, and PDGF BB shows that antiapoptotic markers protect proliferating cells from death by promoting replacement of dead cells by neighboring migrating cells. This is consistent with data obtained by M.M. Kavurma showing that during atherogenesis, apoptotic cell death in the vascular wall is balanced by neighboring phagocytes (the efferocytosis process) with a high clearance of dead cells, thereby limiting the spread of the lesion while maintaining its integrity.^16^

An increase in the amount of the Bax biomarker and a reduction in sFas was observed in initial samples obtained during the primary reconstructions from patients who then had atherosclerosis progression in the late postoperative period. An increase in the size of an atherosclerotic lesion in the postoperative period is associated with more intense apoptotic cell death in the atherosclerotic plaque exacerbated by intraoperative trauma to the vascular wall. This, combined with defective efferocytosis, or due to an excessive pro-inflammatory response, results in apoptotic cells remaining in the intima and eventually undergoing “post-apoptotic” or “secondary” necrosis. Nowadays, secondary necrosis is regarded as an unregulated process in which these apoptotic cells lose their membrane integrity and release all their cellular contents, increasing the inflammatory response and development of atherosclerotic lesions.^17^ This causes increased values of biomarkers p53 and Bax with reduced levels of sFas in samples with progression of atherosclerosis. The values of VEGF A165 and PDGF BB biomarkers were increased in these samples, but not to a statistically significant extent, which gives us reason to assume that the apoptosis system leads to progression of atherosclerosis in the postoperative period. An imbalance in the ratio of the processes of apoptosis and cell proliferation in the future can lead to instability of the atherosclerotic lesion and development of thrombotic complications.

The predictors and features of the pathogenesis of progression of atherosclerotic lesions in the postoperative period identified in this work necessitate the search for a therapeutic effect on the studied biomarkers.

The limitations of our study are the study of biomarkers of apoptosis and cell proliferation by ELISA only, without morphological assessment of the lesions themselves, which is the basis for expanding methodological approaches for further research in the field of PAD. Another limitation is the study of markers of apoptosis and cell proliferation in patients with PAD with stages IIB-III of the disease, according to the Fontaine classification (Rutherford category 3-4), which poses the challenge of expanding the coverage of the study of other stages of the disease.

Currently, this research continues, with further recruitment of patients and expansion of the range of study parameters and types of surgical interventions. We are also searching for predictors of atherosclerosis progression after surgical interventions in patients with PAD based on the values of the markers studied in blood serum. In parallel, a search is underway for correlations between the values of markers of apoptosis, proliferation in the vascular wall in the area of atherosclerotic lesions, and their values in blood serum. In addition, it is also planned to search for drugs that can affect the biomarkers studied.

## CONCLUSIONS

Initially increased levels of the Bax marker against a background of reduced sFas values in the vascular wall in patients with peripheral arterial disease is associated with risk of atherosclerosis progression in the postoperative period.

## Supplementary Material

Supplementary material accompanies this paper:

Clinical Case

This material is available as part of the online article from https://doi.org/10.1590/1677-5449.202200292
