# Supplementary material for: Comprehensive assessment of markers of apoptosis and cell proliferation during progression of atherosclerosis after surgery in patients with peripheral arterial disease
Source: J Vasc Bras. 2023 Feb 10;22:e20220029. doi: 10.1590/1677-5449.202200292 (PMC9925059; doi:10.1590/1677-5449.202200292)
Supplement: Clinical Case [file jvb-22-e20220029-suppl01.pdf]

ORIGINAL ARTICLE

JORNAL VASCULAR BRASILEIRO
ISSN 1677-7301 (Online)

# Comprehensive assessment of markers of apoptosis and cell proliferation during progression of atherosclerosis after surgery in patients with peripheral arterial disease

*Avaliação integral de marcadores de apoptose e proliferação celular na progressão da aterosclerose após cirurgia em pacientes com doença arterial periférica*

Roman Evgenyevich Kalinin[1], Igor Aleksandrovich Suchkov[1], Emma Anatolievna Klimentova[1]

## SUPPLEMENTARY MATERIAL

### Clinical case

A 68-year-old man presented at hospital with a diagnosis of PAD with Leriche syndrome, stage III according to the Fontaine classification. Aortoarteriography and ultrasonography revealed occlusion of the infrarenal aorta and collateral blood flow on both sides along the common femoral, superficial femoral, popliteal, and tibial arteries (Figure S1).

Patient was successfully treated by bifurcation aortofemoral bypass with a synthetic prosthesis (Figures S2 and S3).

In the initial samples of the arterial wall with atherosclerotic lesions, the amounts of proapoptotic markers were Bax: 7.6 ng/mg protein; p 53: 0.3 ng/mg protein; and sFas: 0.1 ng/mg protein. Values of markers of proliferation and migration were PDGF BB: 0.23 ng/mg protein and VEGF A165: 14 pg/mg protein.

After 2 years, the patient was brought to our hospital with pain in the left lower limb. Angiography of the left limb revealed occlusion of the superficial femoral artery with filling of arteries above the knee (Figures S4 and S5).

Subsequently, a femoropopliteal bypass was performed above the knee with a synthetic prosthesis (Figure S6).

During the operation, a section of the vascular wall (superficial femoral artery) with a zone of atherosclerosis progression was taken for subsequent analysis of the values of the study parameters. The levels of the proapoptotic markers were Bax: 9 ng/mg protein; p53: 0.48 ng/mg protein; and sFas: 0.09 ng/mg protein. The values of markers of proliferation and migration were PDGF BB: 0.29 ng/mg protein and VEGF A165: 18.5 pg/mg protein.

Copyright© 2022 The authors. This is an Open Access article distributed under the terms of the Creative Commons Attribution License, which permits unrestricted use, distribution, and reproduction in any medium, provided the original work is properly cited.

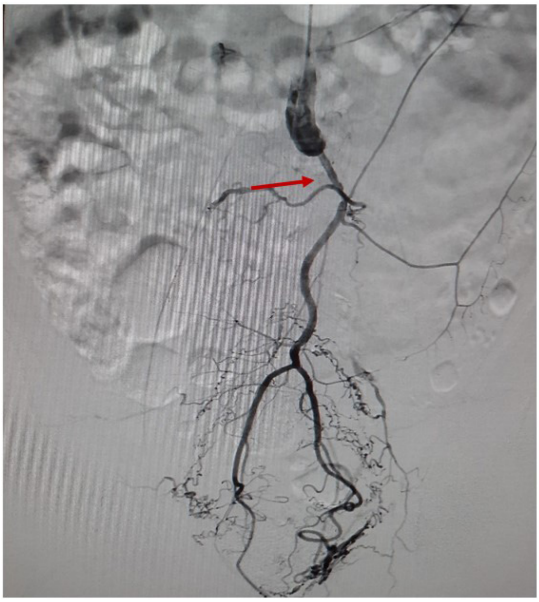

**Figure S1.** Aortoarteriography of the lower extremities. Occlusion of the infrarenal aorta indicated by red arrow.

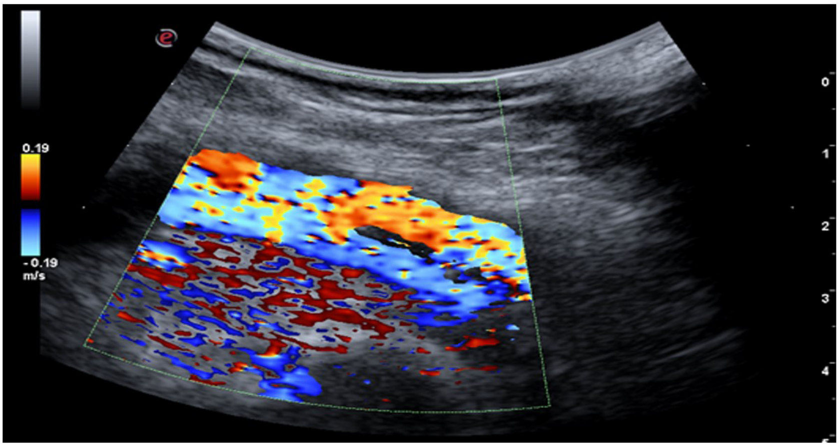


**Figure S2.** Ultrasonography scan. Distal anastomosis of the bifurcation aortofemoral bypass on the left. Color Doppler Mode.

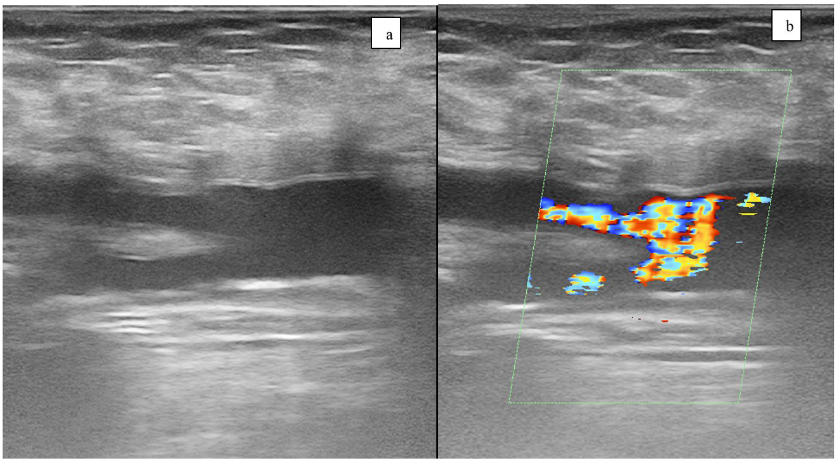


**Figure S3.** Ultrasonography scan. Distal anastomosis of the bifurcation aortofemoral bypass on the left. (a) B-mode; (b) Color Doppler Mode.

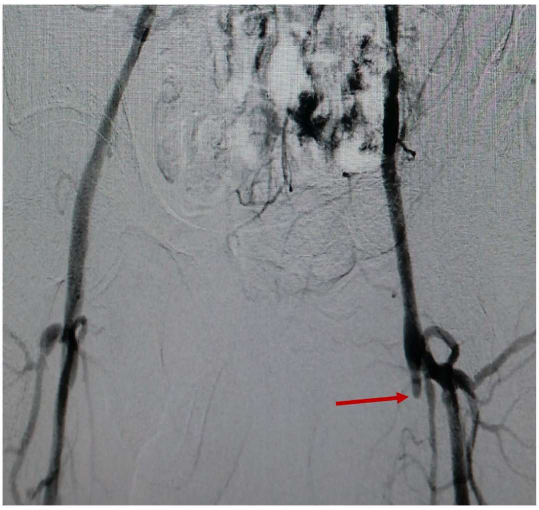

**Figure S4.** Angiography of arteries of the lower extremities. Occlusion of the superficial femoral artery on the left limb indicated by red arrow.

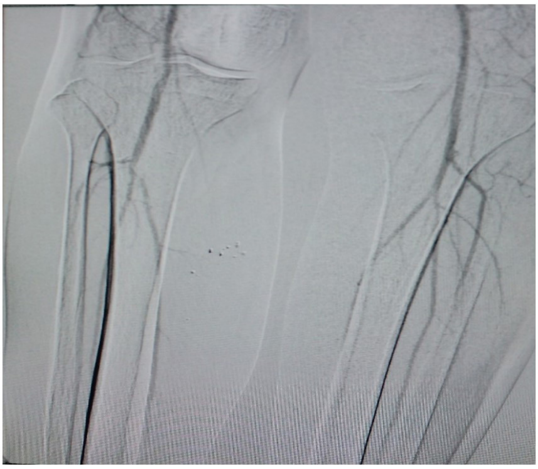

**Figure S5.** Angiography of the arteries of the popliteal segment.

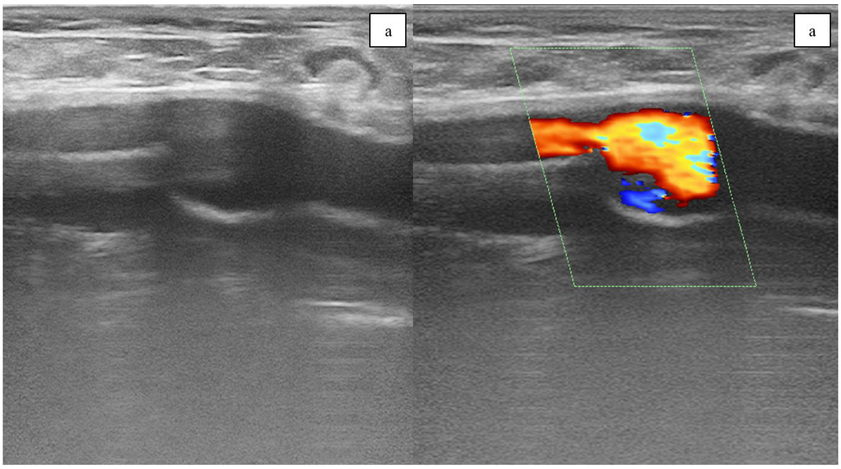


**Figure S6.** Duplex scan. Proximal anastomosis of the femoropopliteal bypass on the left. (a) B – mode; (b) Color Doppler Mode.
